# Terra Firma-Forme Dermatosis or Dermatosis Neglecta: A Diagnostic Continuum Illustrated by a Pediatric Case of Post-circumcision Penile Allodynia

**DOI:** 10.7759/cureus.116238

**Published:** 2026-09-14

**Authors:** Jules Roullée, Guillaume Chaby, Christophe Attencourt, Florian Lombart

**Affiliations:** 1 Dermatology, Centre Hospitalier Universitaire Amiens-Picardie, Amiens, FRA; 2 Pathology, Centre Hospitalier Universitaire Amiens-Picardie, Amiens, FRA

**Keywords:** allodynia, autism spectrum disorder, circumcision, dermatosis neglecta, pediatric dermatology, terra firma-forme dermatosis

## Abstract

Terra firma-forme dermatosis (TFFD) and dermatosis neglecta (DN) can look identical on the skin, both presenting as dirt-like, verrucous plaques that clear with 70% alcohol swabbing. In both conditions, the plaque consists of retained, loosely adherent keratin and debris overlying otherwise normal skin, rather than a true structural epidermal lesion, which is why alcohol removes it. Classically, TFFD persists despite proper washing, whereas DN clears once hygiene resumes; alcohol swabbing does not distinguish between the two. We report a 13-year-old boy with probable autism spectrum disorder who developed a dirt-like, verrucous plaque on the penile shaft after circumcision, where persistent hyperesthesia (increased cutaneous sensitivity) and allodynia (pain provoked by a stimulus that would not normally be painful) made washing painful. Biopsy showed orthokeratotic hyperkeratosis with a normal dermis, ruling out a verrucous epidermal hamartoma. Rubbing the plaque with 70% alcohol produced complete clearance, ruling out a fixed lesion. No findings suggestive of caregiver neglect were identified; the avoidance was localized and self-directed. Hygiene was subsequently discussed with the patient and his caregiver in light of his pain threshold and sensory sensitivities, and he was referred for neuropsychiatric follow-up. This case highlights an underrecognized cutaneous complication of pediatric circumcision, where pain avoidance combined with neurodevelopmental vulnerability can mimic a primary keratinization disorder.

## Introduction

Terra firma-forme dermatosis (TFFD), first described by Duncan WC et al. in 1987, is a benign, acquired disorder of keratinization. It presents as asymptomatic, dirt-like, brown to gray, verrucous plaques that resist conventional washing with soap and water but disappear after vigorous swabbing with 70% isopropyl or ethyl alcohol [1]. It mostly affects children, adolescents, and young adults, with a preference for the neck, ankles, and trunk [2].

Dermatosis neglecta (DN), first described by Poskitt L et al. in 1995 as an “unwashed dermatosis,” looks essentially the same on clinical and dermoscopic examination, and largely the same under the microscope, but it arises from localized avoidance of hygiene rather than from a primary keratinization defect [3]. Reported causes of DN include pain, localized hyperesthesia (increased cutaneous sensitivity) or allodynia (pain provoked specifically by a normally non-painful stimulus, such as touching or washing), immobilization, prior surgery or trauma, and psychiatric or neurodevelopmental conditions that make self-care difficult [4]. Whether TFFD and DN are truly separate entities or simply the same process with two different names, distinguished mainly by cause, is still debated [5].

What the two share is a common substrate: in both, the plaque is made of retained, loosely adherent keratin and debris sitting on top of otherwise normal skin. This explains why alcohol clears them, helping to dissolve and mechanically remove the accumulated material, whereas a fixed structural lesion cannot be removed in this way. It also explains their dermoscopic appearance. Dermoscopy, a non-invasive magnified examination using polarized light or a liquid interface to reduce surface reflection, typically shows brown clods arranged in a cobblestone-like pattern in both conditions, reflecting surface debris accumulation rather than a true pigment network, vascular structures, or a proliferative growth pattern.

Genital involvement of either condition is uncommon, though a handful of pediatric penile cases of TFFD have already been reported [6,7]. In this location, the differential diagnosis includes condyloma acuminatum, usually the first consideration for a verrucous penile plaque in an adolescent, and verrucous epidermal hamartoma (epidermal nevus), a benign but structurally fixed overgrowth of the epidermis that, unlike TFFD or DN, cannot be rubbed away. We report a case in a 13-year-old boy with a probable autism spectrum disorder in whom the plaque developed after circumcision, in the setting of postsurgical allodynia, and we discuss how the two diagnoses overlap and how they can, in practice, be distinguished.

## Case presentation

A 13-year-old boy followed in neuropediatrics for dyslexia, dysorthographia, and a probable but still unlabeled autism spectrum disorder was referred to dermatology for a large, dirt-like plaque of the penis. He had been circumcised for phimosis 18 months earlier and had developed persistent hyperesthesia and allodynia of the penile skin thereafter, which made washing that area painful and led him to avoid it. The plaque became visible approximately four months after this avoidance began. On examination, light touch of the penile skin was sufficient to provoke pain, confirming the allodynia clinically; the dermatological examination itself had to be conducted slowly and with the patient's cooperation.

The patient managed his own hygiene independently, consistent with expectations for his age, and was afforded a corresponding degree of privacy while washing. He himself reported the growing lesion to his caregiver, which prompted the consultation. No history or clinical finding suggestive of caregiver neglect was identified: the family was engaged and supportive throughout and consistently attended scheduled medical appointments, and the privacy afforded during hygiene reflected age-appropriate autonomy rather than inattention. The presentation was therefore considered consistent with localized, self-directed hygiene avoidance secondary to postsurgical allodynia rather than broader caregiver neglect.

On examination, a large, well-demarcated, pigmented, verrucous keratotic plaque (approximately 6 × 3 cm) covered the lateral and dorsal aspects of the penile shaft and extended toward the coronal margin, with the glans mucosa spared (Figure 1).

**Figure 1:**
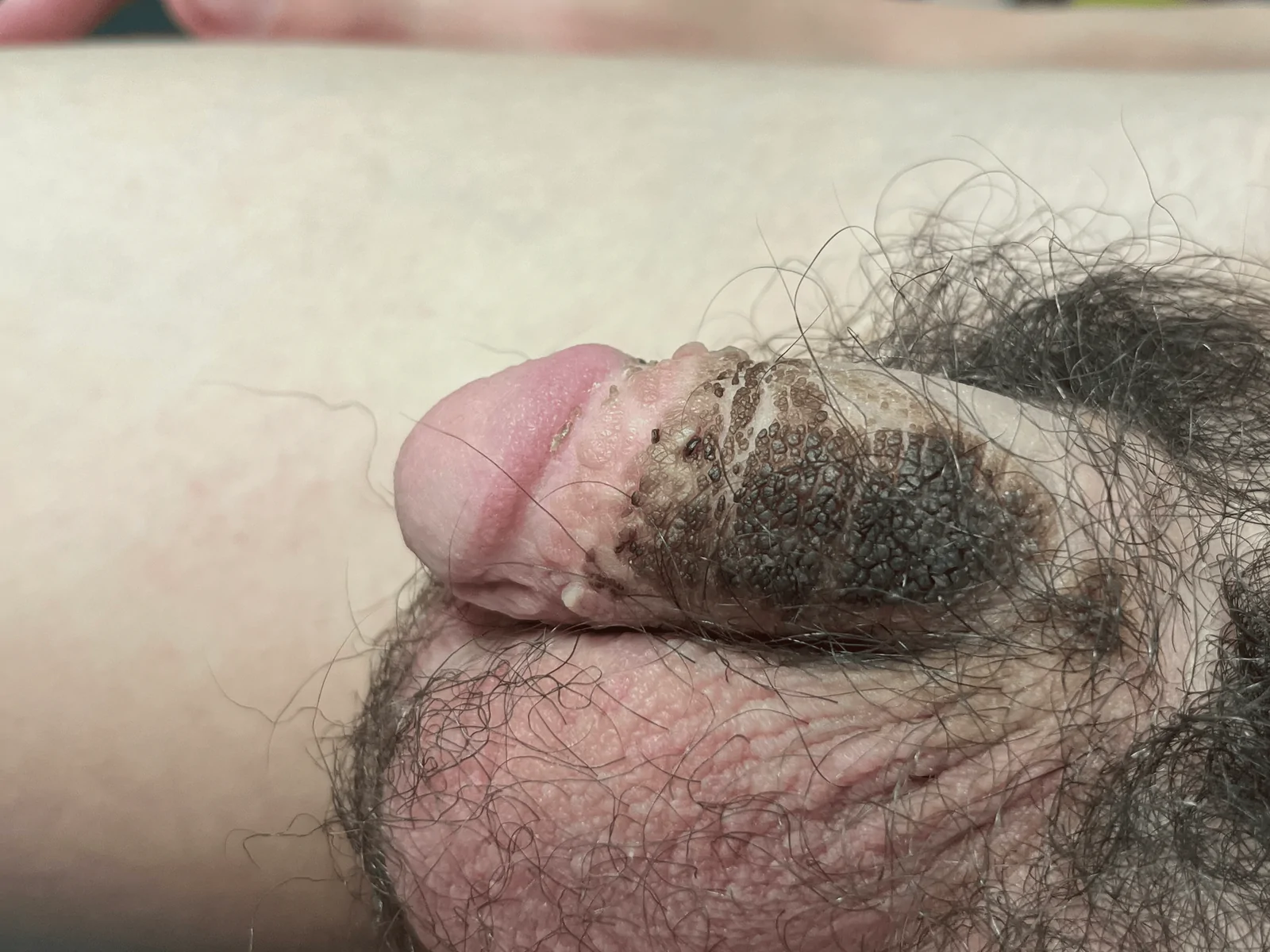
Verrucous pigmented plaque of the penile shaft. Initial clinical presentation showing a large (6 × 3 cm), well-demarcated, pigmented, verrucous keratotic plaque involving the lateral and dorsal aspects of the penile shaft and extending toward the coronal margin, with sparing of the glans mucosa.

Dermoscopy was performed using a polarized handheld dermoscope at ×10 magnification. It showed multiple brown to dark-brown clods arranged in a cobblestone pattern over otherwise unremarkable skin (Figure 2), a pattern suggestive of retained surface keratin and debris rather than a structural lesion. No dotted vessels, which can be seen in verrucous epidermal hamartoma, were visible. This did not formally exclude a hamartoma, however, since a superficial layer of accumulated debris may itself obscure any underlying vascular structures. A biopsy was therefore performed to exclude a verrucous epidermal hamartoma, given the verrucous appearance of the plaque and its onset around puberty.

**Figure 2:**
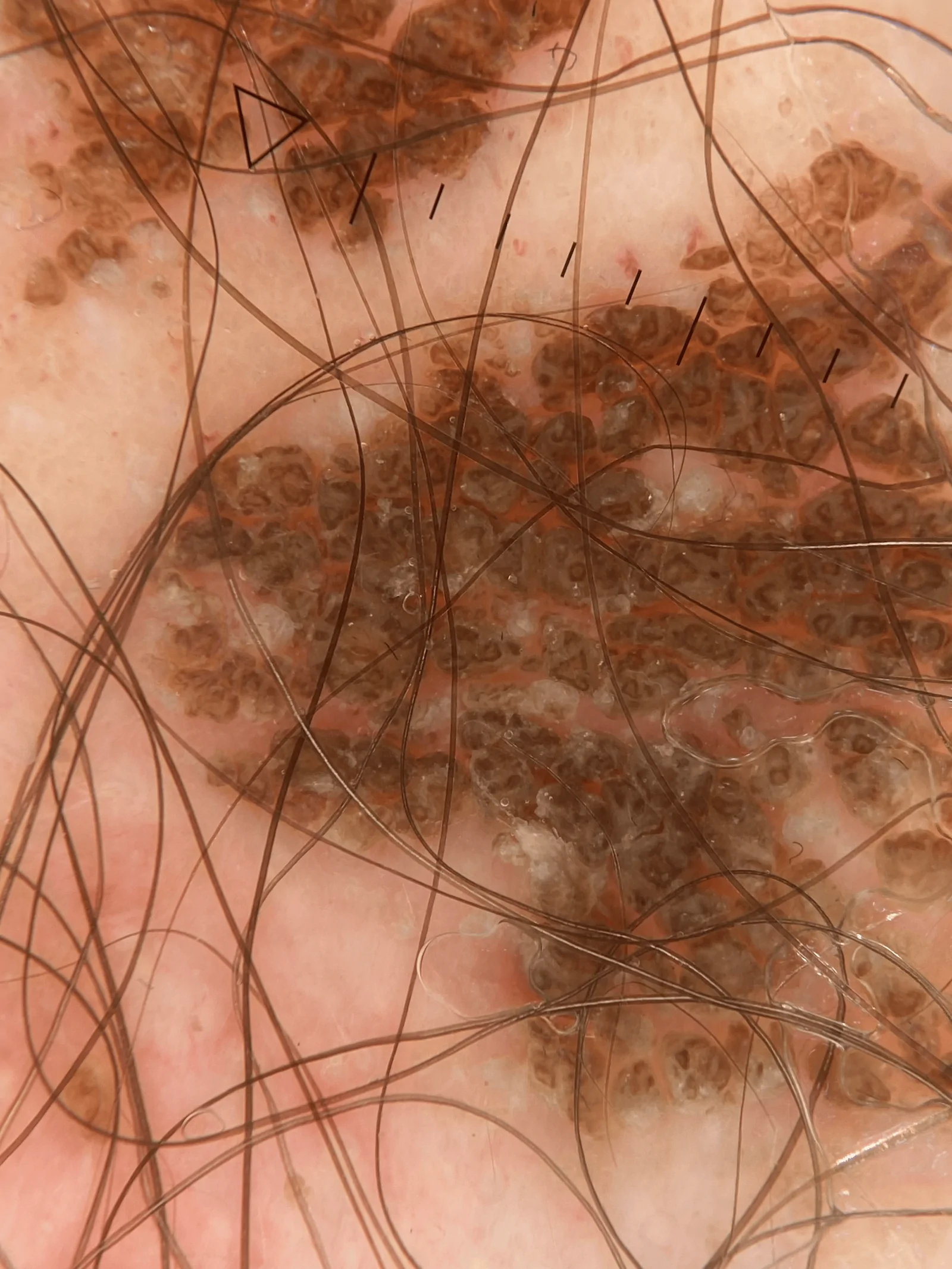
Dermoscopic cobblestone pattern of brown clods. Dermoscopic view of the plaque (polarized handheld dermoscope, ×10) showing multiple brown to dark-brown clods arranged in a cobblestone-like pattern over otherwise unremarkable skin. The black lines and arrowhead visible in the image correspond to the dermoscope's built-in measurement scale and do not indicate a region of interest.

Histology showed orthokeratotic hyperkeratosis (excess but normally maturing keratin, without cellular atypia), compact and whorled in places but mostly lamellar and basket-woven, with mild papillomatosis of the epidermis and an unremarkable dermis (Figure 3). Periodic acid-Schiff staining (PAS) staining was positive, highlighting abundant yeast forms consistent with *Pityrosporum* trapped within the keratin. No koilocytosis or other human papillomavirus (HPV)-associated cytopathic change was identified, excluding condyloma. These findings fit equally well with TFFD or DN, and the two could only be distinguished based on the clinical picture.

**Figure 3:**
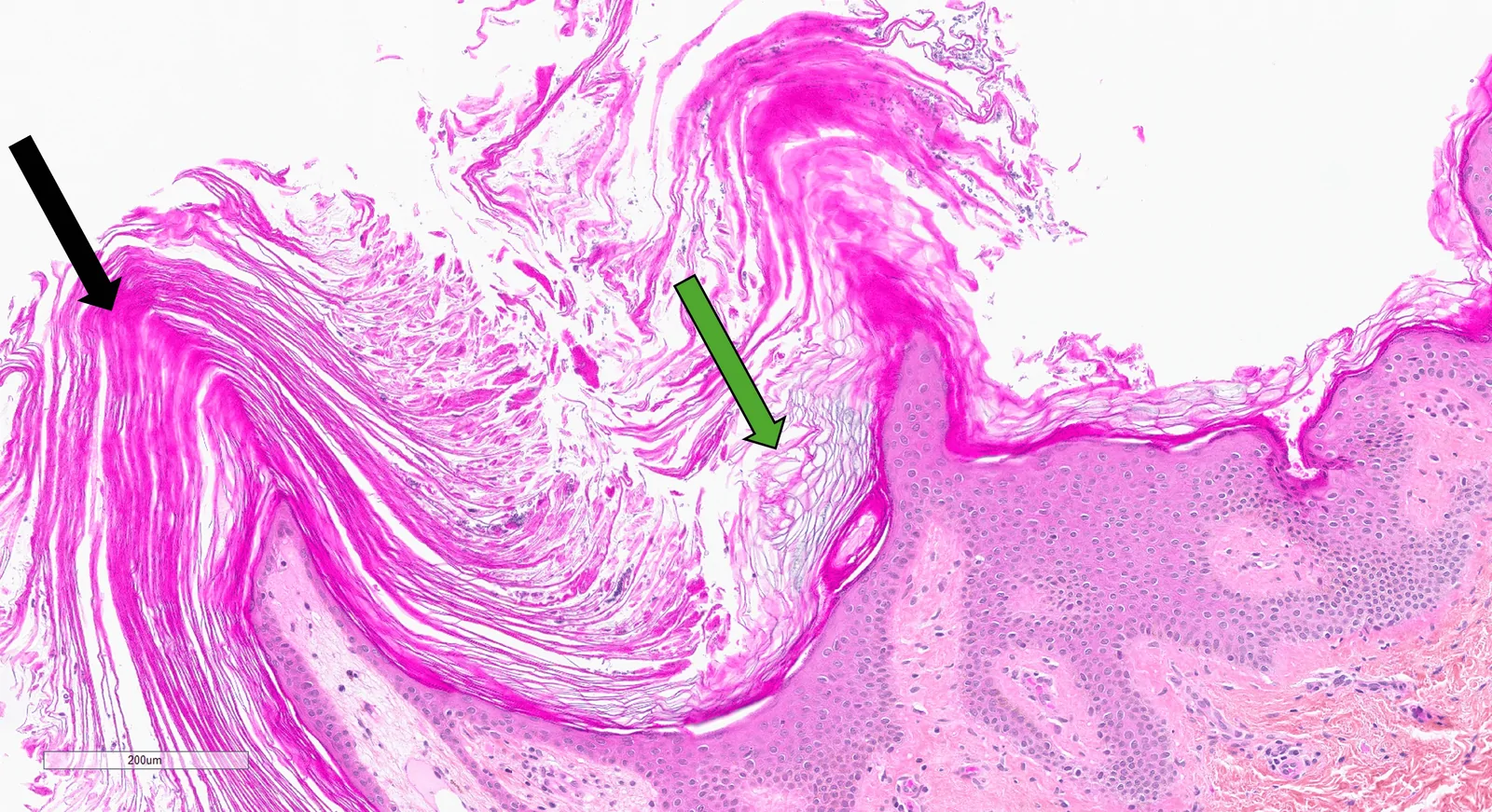
Orthokeratotic hyperkeratosis on histology. H&E-stained histopathological section (scale bar = 200 μm) showing orthokeratotic hyperkeratosis, predominantly lamellar (black arrow) and basket-woven (green arrow), overlying a mildly papillomatous epidermis.

The patient was reviewed at a follow-up consultation once the histological results were available. The findings argued against a verrucous epidermal hamartoma and prompted us to attempt an alcohol wipe test at that visit. Firm rubbing of the plaque with gauze soaked in 70% isopropyl alcohol for approximately three minutes cleared it completely (Figure 4), confirming the absence of a fixed structural lesion. Alcohol swabbing does not distinguish between TFFD and DN, however, since both clear equally well with alcohol.

**Figure 4:**
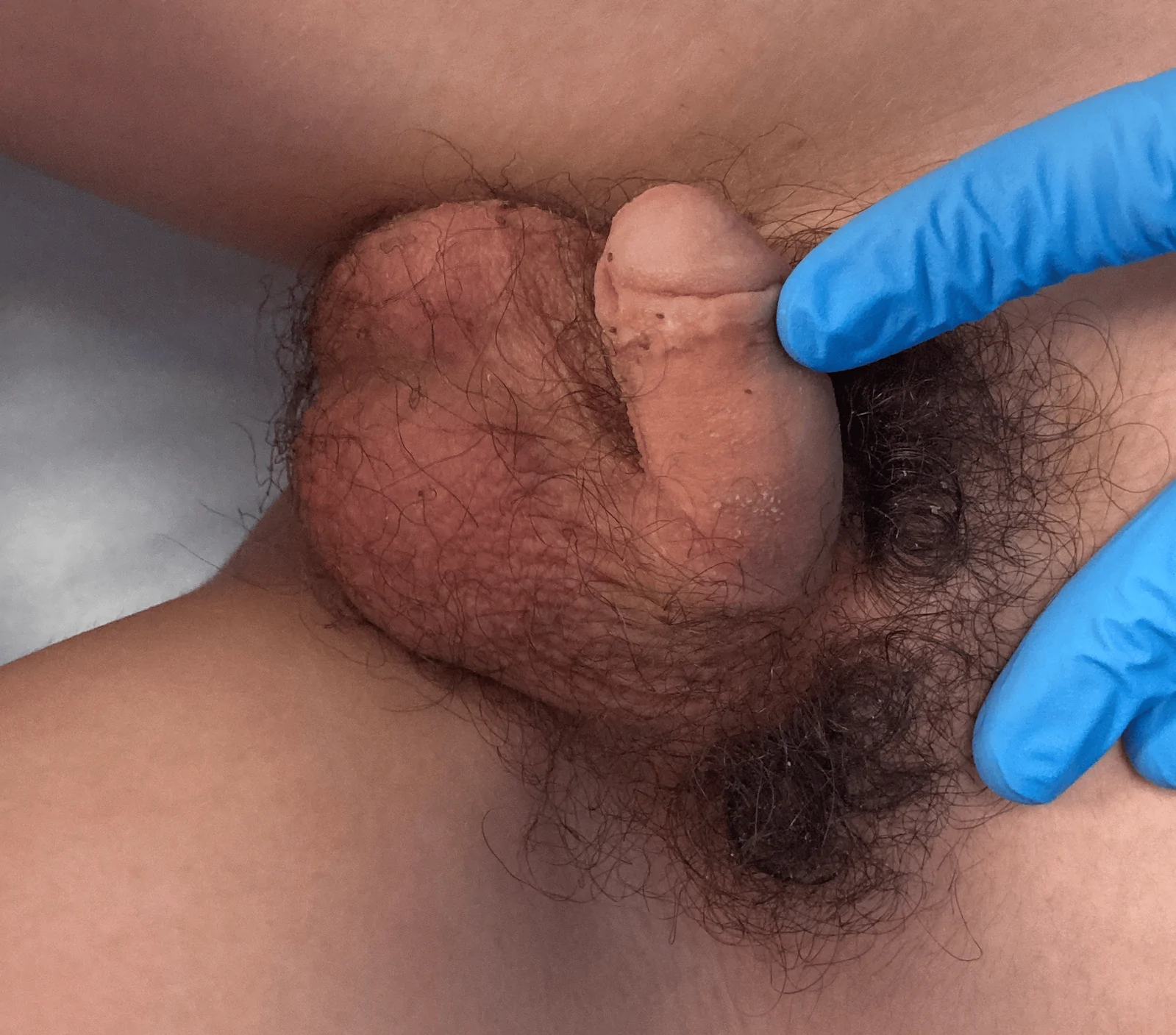
Complete clearance after alcohol swab test. Complete clearance of the plaque immediately after firm rubbing with a gauze pad soaked in 70% isopropyl alcohol, ruling out a fixed structural lesion such as an epidermal hamartoma, though this test does not on its own distinguish TFFD from DN. TFFD: Terra firma-forme dermatosis; DN: Dermatosis neglecta.

Hygiene technique was then discussed and demonstrated to the patient, taking into account his pain threshold and sensory sensitivities. The mechanism was explained to both the patient and his mother, emphasizing that the lesion was retained debris rather than a growth and that pain-driven avoidance rather than carelessness had caused it, so that hygiene could be resumed without stigma. He was referred for neuropsychiatric follow-up. No structured desensitization protocol or occupational therapy intervention was implemented. A telephone follow-up seven months after the consultation confirmed that the plaque had not recurred.

## Discussion

This case shows how much TFFD and DN can overlap. Both look the same to the naked eye and on dermoscopy, with dirt-like, verrucous, hyperpigmented plaques and a brown clod pattern, and both clear completely with a 70% alcohol wipe test [1,8]. Histology does not separate them well either: orthokeratotic, lamellar or basket-woven hyperkeratosis with mild papillomatosis and a normal dermis, as seen in our patient, has been described in both conditions [2,9]. The keratin also contained abundant yeast forms consistent with *Pityrosporum*, which is unsurprising: skin that stays unwashed and occluded for months favours *Malassezia* overgrowth, and this fits a picture of hygiene avoidance better than a primary, otherwise sterile disorder of keratinization.

In our patient, the distinction between the two diagnoses is not just theoretical, and the chronology tells its own story. The plaque appeared only after circumcision, in an area the procedure had left painful and hyperesthetic. He also has a probable autism spectrum disorder, independently associated with difficulties in self-care and hygiene, particularly around areas of the body that are painful or sensory-sensitive [10]. By that age, he was expected to wash himself, so his difficulty went unnoticed. Together, a new painful trigger and a pre-existing vulnerability to hygiene disruption give a coherent explanation that a primary keratinization disorder alone does not provide, and we therefore lean toward DN secondary to post-circumcision allodynia.

This case also illustrates a distinction worth keeping in mind when the term “dermatosis neglecta” is applied to a pediatric patient. Although the name may suggest inadequate caregiving, the avoidance may be localized and self-directed rather than reflecting a broader failure of care, as it was here, in a boy whose hygiene was age-appropriately autonomous and whose psychosocial context was otherwise unremarkable. This does not exclude the possibility that neglect by others contributes in other cases, and a presentation of this kind in a child should still prompt the clinician to consider both explanations rather than assuming either.

This report has several limitations. The wash-persistence test that classically distinguishes TFFD from DN, TFFD persisting despite thorough washing while DN clears once ordinary hygiene resumes, was not performed, since the plaque had already cleared with alcohol; the diagnosis of DN therefore rests on clinical and behavioural inference. The alcohol wipe test served simultaneously as diagnostic confirmation and as treatment, so no gradual, hygiene-driven resolution was observed. Absence of recurrence at seven months was established by telephone rather than by clinical examination. Finally, no PAS-stained image was available to document the yeast finding.

## Conclusions

Genital involvement of TFFD or DN is uncommon and can be mistaken for more concerning verrucous or hamartomatous lesions, particularly around puberty. In children with postsurgical allodynia combined with neurodevelopmental conditions that affect self-care, as in the present case where both factors coexisted, localized hygiene avoidance is a plausible explanation for dirt-like, resistant, hyperpigmented plaques and favors DN over a primary keratinization disorder, though this rests on clinical and behavioral inference rather than on the classic confirmatory wash-persistence test. A simple 70% alcohol wipe test doubles as treatment and points toward these two diagnoses rather than a fixed lesion. In our patient, the biopsy preceded the alcohol wipe test. In retrospect, performing the wipe test first might have spared this child an unnecessary procedure, and we would recommend that sequence to clinicians facing a similar presentation.
